# Solubility and Physical Stability Enhancement of Loratadine by Preparation of Co-Amorphous Solid Dispersion with Chlorpheniramine and Polyvinylpyrrolidone

**DOI:** 10.3390/pharmaceutics15112558

**Published:** 2023-10-31

**Authors:** Krit Suknuntha, Nattakanwadee Khumpirapang, Vimon Tantishaiyakul, Siriporn Okonogi

**Affiliations:** 1 Drug Delivery System Excellence Centre, Faculty of Pharmaceutical Sciences, Prince of Songkla University, Songkhla 90110, Thailand; krit@pharmacy.psu.ac.th (K.S.); vimon.t@psu.ac.th (V.T.); 2 Department of Pharmaceutical Chemistry, Faculty of Pharmaceutical Sciences, Prince of Songkla University, Songkhla 90110, Thailand; 3 Department of Pharmaceutical Chemistry and Pharmacognosy, Faculty of Pharmaceutical Sciences, Naresuan University, Phitsanulok 65000, Thailand; nattakanwadeek@nu.ac.th; 4 Department of Pharmaceutical Sciences, Faculty of Pharmacy, Chiang Mai University, Chiang Mai 50200, Thailand; 5 Center of Excellence in Pharmaceutical Nanotechnology, Faculty of Pharmacy, Chiang Mai University, Chiang Mai 50200, Thailand

**Keywords:** loratadine, chlorpheniramine, polyvinylpyrrolidone, solubility enhancement, solid dispersion, amorphous, quench cooling, film casting

## Abstract

Loratadine (LRD), a non-sedating and slow-acting antihistamine, is often given in combination with short-onset chlorpheniramine maleate (CPM) to increase efficacy. However, LRD has poor water solubility resulting in low bioavailability. The aim of this study was to improve LRD solubility by preparing co-amorphous LRD-CPM. However, the obtained co-amorphous LRD-CPM recrystallized rapidly, and the solubility of LRD returned to a poor state again. Therefore, co-amorphous LRD-CPM solid dispersions using polyvinylpyrrolidone (PVP) as a carrier were prepared. The obtained solid dispersions were characterized using X-ray powder diffraction (XRPD), differential scanning calorimetry (DSC), and Fourier transform infrared spectroscopy (FT-IR). The solubility, dissolution, and mechanism of drug release from the LRD-CPM/PVP co-amorphous solid dispersions were studied and compared with those of intact LRD, LRD/PVP solid dispersions, and co-amorphous LRD-CPM mixtures. The results from XRPD and DSC confirmed the amorphous form of LRD in the co-amorphous solid dispersions. The FTIR results indicated that there was no intermolecular interaction between LRD, CPM, and PVP. In conclusion, the obtained LRD-CPM/PVP co-amorphous solid dispersions can successfully increase the water solubility and dissolution of LRD and extend the amorphous state of LRD without recrystallization.

## 1. Introduction

Oral administration is the most preferable route for drug delivery because of minimal invasiveness, ease of use, smaller unit, and ease of production [1,2]. Most new drugs or active pharmaceutical ingredients (APIs) are poorly water-soluble drugs and have low bioavailability after oral administration. For this reason, enhancing the solubility and dissolution of drugs is an important strategy for improving drug bioavailability [3]. The dispersion of the solid state of poorly water-soluble APIs in inert amorphous or crystalline hydrophilic polymer matrices, known as solid dispersions, has been reported for enhancing the dissolution rate and oral bioavailability [3,4,5]. The mechanisms of solid dispersion to enhance the dissolution rate of APIs can be described by several strategies such as eutectic mixture formation, increasing wettability by mixing with hydrophilic carriers, amorphous form formation, or crystallinity reduction [3]. The amorphous state of drugs can be formed, but the interaction energy of the amorphous form is high and drugs may crystallize [4]. Inhibition of crystallization can be achieved by adding a high amount of an inert carrier, increasing the strength of drug–polymer interaction, and reducing the mobility of the system [6,7]. In addition, to improve the amorphous stability, blending with two polymers can be used for a synergistic effect [8,9,10]. Amorphous drugs incorporated between polymer chains can be named a polymeric amorphous solid dispersion [11].

A new strategy for the solid dispersion technique is the use of a co-amorphous system [11]. A co-amorphous system is defined as multiple components with a single amorphous phase with or without intermolecular interaction between the components [12,13]. This system is a combination of two or more small molecules which may a drug–drug [14] or drug–amino acid [15] combination instead of polymers. A co-amorphous system can be administered as a combination of therapeutic drugs or natural compounds [12,13,16,17]. A drug–drug co-amorphous system offers the new development of new combination therapy that enhances the solubility and dissolution of poorly water-soluble drugs. Polymers such as polyvinylpyrrolidone (PVP) can be added to a drug–drug or drug–amino acid co-amorphous system to further increase the solubility and stability of the amorphous system [15].

Loratadine (LRD) is a second-generation histamine H1 receptor antagonist (antihistamine) developed to reduce sedation and anticholinergic effects. The recommended dose of LRD is 10 mg once daily. The drawback of LRD is that it has an unpredictable onset and does not have a strong correlation with serum concentration. In some cases, the onset may begin 4 h after oral administration [18]. LRD is classified under the biopharmaceutical classification system as a class II drug due to its low solubility [19]. Due to these poor properties, an attempt was made to increase the solubility and dissolution rate of LRD using solid dispersions [20,21,22,23]. While the first generation of antihistamines like chlorpheniramine maleate (CPM) can present a short onset, sedation is an adverse effect [24]. The usual adult dose for allergic rhinitis is a 4 mg oral tablet every 4 to 6 h. Therefore, the combination of non-sedating LRD with a sedating CRM demonstrates greater efficacy for allergic symptoms than LRD alone. These combinations provide 24 h antihistamine efficacy with a short onset of action and low side effects [25].

In this study, co-amorphous solid dispersions of LRD-CPM with PVP were prepared by the film casting method. The solubility and dissolution profiles of LRD in solid dispersions with different concentrations of PVP were investigated. The glass transition temperature (Tg) was observed using differential scanning calorimetry (DSC) and reflected the stability of the co-amorphous system with PVP. The intermolecular interactions of the co-amorphous system and PVP were studied using Fourier transform infrared spectroscopy (FT-IR). Moreover, the amorphous stability of solid dispersions was also investigated using X-ray powder diffraction (XRPD).

## 2. Materials and Methods

### 2.1. Materials

**Figure 1:**
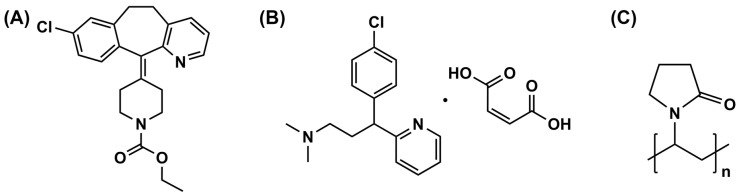
Chemical structure of LRD (**A**), CPM (**B**), and PVP (**C**).

LRD was obtained from Vasudha Pharma Chem (Vishakapatnam, India). PVP (K-90) with an average molecular weight of 1,100,000 was kindly supplied by BASF, Bangkok, Thailand. CPM was kindly supplied by Pharmasan Labs, Nonthaburi, Thailand. All other reagents were of analytical grade. The chemical structures of LRD, CPM, and PVP are shown in Figure 1.

### 2.2. Preparation of Amorphous and Co-Amorphous Materials

The amorphous form of LRD was prepared by the quench cooling method. Approximately 100 mg of LRD was added to an aluminum pan. The drug powder was heated using a hot plate (IKA, Staufen, Germany) until it became liquid. The aluminum pan was removed from the hot plate, and liquid nitrogen was immediately poured into it. The recommended doses of LRD and CPM were 10 mg/tablet and 4 mg/tablet, respectively, and were selected for co-amorphous preparation. Therefore, the weight ratio of LRD:CPM used in this study was 10:4. For the preparation of the co-amorphous system, accurate weights of LRD (10 mg) and CPM (4 mg) were mixed and dissolved in 10 mL of ethanol; subsequently, the mixture solution was cast on 8 × 8 cm^2^ mold and dried in a hot air oven at 60 °C for 8 h. The amorphous and co-amorphous powder samples were collected in a screw-cap glass bottle and stored in a desiccator for further studies.

### 2.3. Preparation of Solid Dispersions and Physical Mixtures

The co-amorphous solid dispersions of LRD-CPM/PVP were prepared as follows. Accurate weights of LRD (10 mg) and CPM (4 mg) were mixed with PVP at different weight ratios of LRD-CPM/PVP = 1/9, 3/7, and 5/5 in a 50 mL polyethylene terephthalate tube. Then, 10 mL of ethanol was added, and the mixture was mixed using a reciprocating shaker until a homogeneous solution was obtained. The mixture solutions with the equivalent of 10 mg LRD were cast on an 8 × 8 cm^2^ mold and dried in a hot air oven at 60 °C for 8 h to ensure that all residual solvent was removed. All film samples were stored in a screw-cap glass bottle in a desiccator cabinet. The stored film samples were gently ground using a mortar and pestle with the aid of liquid nitrogen to a fine powder prior to further studies. At the same time, the amorphous solid dispersions of LRD/PVP and CPM/PVP were also prepared in the same manner as the co-amorphous solid dispersions of LRD-CPM/PVP as mentioned above. The physical mixtures of LRD/PVP or LRD-CPM/PVP at all ratios of drug to carrier were prepared by gently mixing the accurate weight of drugs and PVP without the use of ethanol.

### 2.4. Solubility Studies

The solubility of LRD of each system compared to the physical mixture was determined by immersion of each sample equivalent to 50 mg of LRD into 30 mL of enzyme-free USP simulated intestinal fluid at pH 6.8. The mixture was continuously well rotated using a reciprocating shaker for 48 h. An aliquot of 1.0 mL of the sample suspension was withdrawn and filtered through a 0.45 µm PVDF syringe filter. LRD content was analyzed using reverse-phase high-performance liquid chromatography (HPLC). All measurements were performed in triplicate.

### 2.5. DSC Analysis

DSC measurements were performed using a DSC 8000 (Perkin-Elmer, Waltham, MA, USA). The sample (2.0–8.0 mg) was accurately weighed and placed in a crimped aluminum pan. The glass transition temperature (Tg) of the amorphous sample was measured using a heat–cool cycle by heating the samples from 50 °C to 140 °C at a heating rate of 10 °C/min. Then, the samples were cooled to 50 °C at a cooling rate of 10 °C/min and reheated to 250 °C at a heating rate of 10 °C/min. The Tg value is the midpoint of the transition phase in the second heating cycle.

### 2.6. XRPD Analysis

The XRPD analysis was performed using a Philips PW 3710 diffractometer (PANalytical, Almelo, the Netherlands) with Cu-Kα radiation, collimated by a 0.08° divergence slit and a 0.2° receiving slit and scanning at a rate of 2.4°/min over the 2θ range of 5.0–60.0°. The XRPD patterns of all samples were observed on days 0, 30, 90, 180, and 360. All diffractograms were analyzed using X’Pert HighScore Plus software version 2.2.2.

### 2.7. FT-IR Analysis

FT-IR spectra were measured on a Fourier transform infrared spectrometer (Perkin-Elmer, Waltham, MA, USA). All samples were prepared as pellets using KBr. Spectra were recorded from 4400 to 450 cm^−1^ by averaging 16 scans at a resolution of 4 cm^−1^. All spectra were analyzed using the PerkinElmer Spectrum software package for Windows version 5.02.

### 2.8. Dissolution Studies

The release profile of LRD from each system was measured in simulated intestinal fluid pH 6.8 at 37 ± 0.5 °C using a VK 7000 dissolution tester in conjunction with an autosampler (Vankel Industries, Edison, Atlanta, NJ, USA) with a USP apparatus 2. Samples equivalent to 10 mg LRD were put into a flask containing 900 mL of release medium with a paddle spin of 50 rpm. Then, 5 mL of sample solution was collected and filtered using a 0.22 µm inline filter at regular intervals of 2, 4, 6, 8, 10, 20, 30, 60, 90, 120, 180, 240, and 360 min. An equal amount of fresh medium at the same temperature was replaced after each sample collection to maintain sink conditions. The drug concentration of each sample was determined using HPLC. All measurements were performed in triplicate.

The release kinetics of LRD from the solid dispersions were analyzed based on the correlation coefficients (r^2^) of first-order kinetics (Equation (1)), Higuchi (Equation (2)), Korsmeyer–Peppas (Equation (3)), and Hixson–Crowell (Equation (4)) dissolution models employing the following equations [26]:

(1) $$\ln\left(\frac{M_{0}}{M_{t}}\right)=\mathrm{kt},$$

(2) $$M_{t}=k\sqrt{t},$$

(3) $$\frac{M_{t}}{M_{\infty }}={\mathrm{kt}}^{n},$$

(4) $$M_{0}^{1/3}-M_{t}^{1/3}=\mathrm{kt}$$

where M_0_, M_t_, and M_∞_ are the amounts of drug released at time zero, time t, and infinite time, respectively; k is the release constant; and n is the release exponent.

### 2.9. HPLC Analysis

LRD quantitative analysis was performed by a validated HPLC method using a Hitachi HPLC system (Hitachi, Tokyo, Japan) [27]. A C18 reverse-phase column (Thermo, Waltham, MA, USA; 4.6 mm × 15 cm; 5 µm) was used. A mixture of 0.01 M dibasic potassium phosphate, chromatography-grade methanol, and chromatography-grade acetonitrile at a ratio of 7:6:6 was used for the mobile phase. The pH of the mobile phase was adjusted to 7.2 using a 10% phosphoric acid solution. The flow rate was 1.0 mL/min, and the injection volume was 20 µL. The column temperature was maintained at ambient temperature. A UV-Vis diode array was used to detect LRD at a wavelength of 254 nm.

### 2.10. Statistical Analysis

Analysis of variance (ANOVA) of the results was performed using IBM SPSS version 22.0 for Windows (IBM, New York, NY, USA). A post hoc test (*p* < 0.05) of multiple comparisons was performed using Tukey’s test to determine the mean difference of the obtained data.

## 3. Results

### 3.1. Outer Appearance of the Samples

The freshly prepared amorphous LRD and co-amorphous LRD-CPM appeared as transparent solid masses. After gently grinding each sample, a transparent powder with low physical stability was obtained. Most of the transparent amorphous LRD powder transformed to an opaque white powder within 1 h, indicating rapid recrystallization. For the co-amorphous LRD-CPM, recrystallization occurred gradually, and white opaque powder was observed within 30 days.

For the amorphous and co-amorphous solid dispersions, transparent and smooth films were obtained from LRD/PVP and LRD-CPM/PVP systems, respectively, at all weight ratios. After gently grinding each sample, a transparent power was obtained, and no opaque white powder was obtained. This indicated no recrystallization had occurred.

### 3.2. Solubility of LRD

The solubility of LRD from various systems in enzyme-free USP simulated intestinal fluid at pH 6.8 is shown in Table 1. It was found that the LRD/PVP solid dispersion showed slightly higher solubility than the crystalline LRD and the physical mixture. The solubility of the LRD-CPM/PVP solid dispersion at all ratios was greater than that of the crystalline LRD, the physical mixture, and the LRD/PVP solid dispersion. It was noted that the solubility of LRD in solid dispersions depended on the polymer concentration. Increasing the PVP ratio increased the solubility of LRD. At drug-to-polymer ratios of 3/7 and 1/9, the highest solubility of LRD was obtained.

### 3.3. DSC Analysis

**Figure 2:**
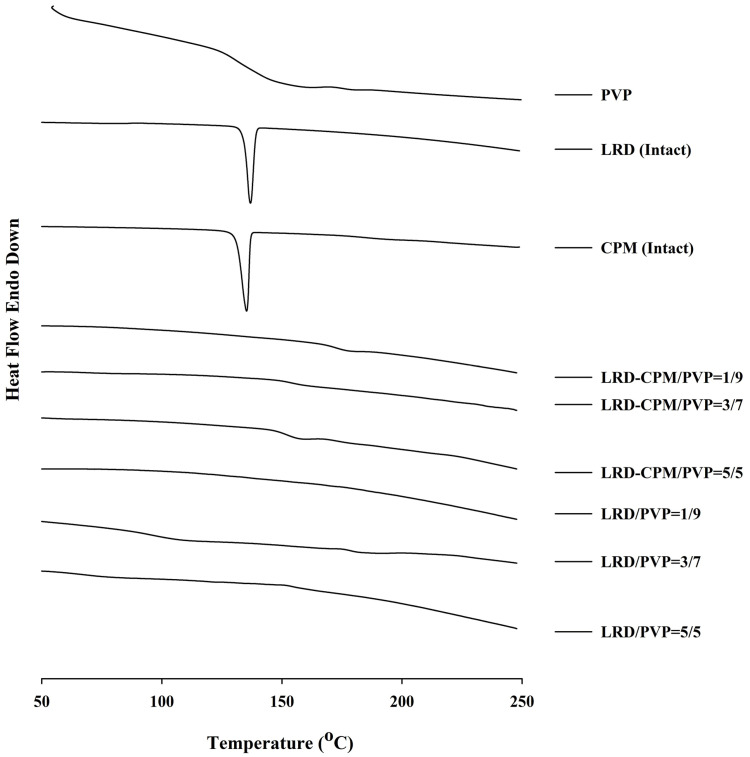
DSC thermograms of the amorphous LRD and co-amorphous LRD-CPM solid dispersions in comparison with the intact drugs and PVP.

The DSC thermograms of each sample are shown in Figure 2. Endothermic peaks indicating melting points of the intact LRD and CPM were found at 136.89 °C and 135.27 °C, respectively. The thermogram of PVP showed a broad endothermic range from 100 °C to 150 °C, indicating water loss due to the high hygroscopicity of PVP. In contrast to the solid dispersions of LRD/PVP and LRD-CPM/PVP, no endothermic peaks were shown at 136.89 °C and 135.27 °C, indicating that the drug dispersion was in an amorphous or solid solution state. The observed Tg values for LRD/PVP and LRD-CPM/PVP solid dispersions are listed in Table 2.

### 3.4. XRPD Analysis

**Figure 3:**
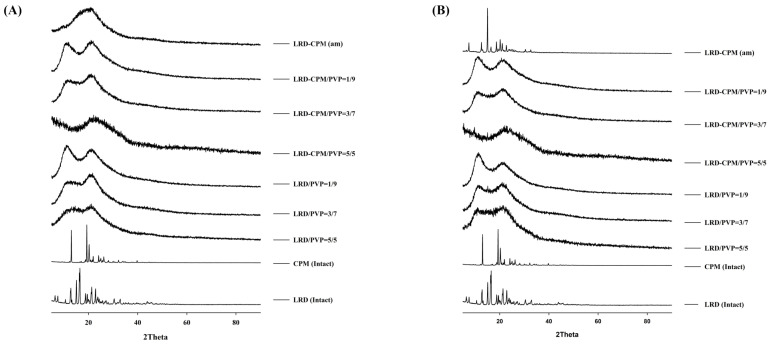
XRPD patterns of the amorphous LRD and co-amorphous LRD-CPM solid dispersions in comparison with the intact drugs and co-amorphous mixture of LRD-CPM (am) after being freshly prepared (**A**) and kept for 30 days (**B**).

XRPD was performed to confirm the amorphous or crystalline state of the samples. As shown in Figure 3, the XRPD patterns of the intact LRD and CPM showed sharp identical peaks, which confirmed the crystallinity form of the drugs. The characteristic diffraction peaks of LRD were observed at 2θ of 6.4, 7.5, 10.6, 12.8, 15.1, 16.2, 16.5, 18.7, 19.5, 21.1, 21.3, 22.9, 23.8, 30.4, and 32.9° with three strong peaks at 15.1, 16.2, and 16.5°, corresponding to LRD polymorphic form A previously characterized in US patent 6335347B1 [28]. CPM showed characteristic diffraction peaks at 2θ of 13.0, 19.3, 20.2, 21.9, 24.1, and 26.2° with three strong peaks at 13.0, 19.3, and 20.2°. The freshly prepared co-amorphous LRD-CPM and the solid dispersions of LRD/PVP and co-amorphous LRD-CPM/PVP of all ratios of drug to carrier showed halo patterns without any crystalline peaks, as shown in Figure 3A.

**Figure 4:**
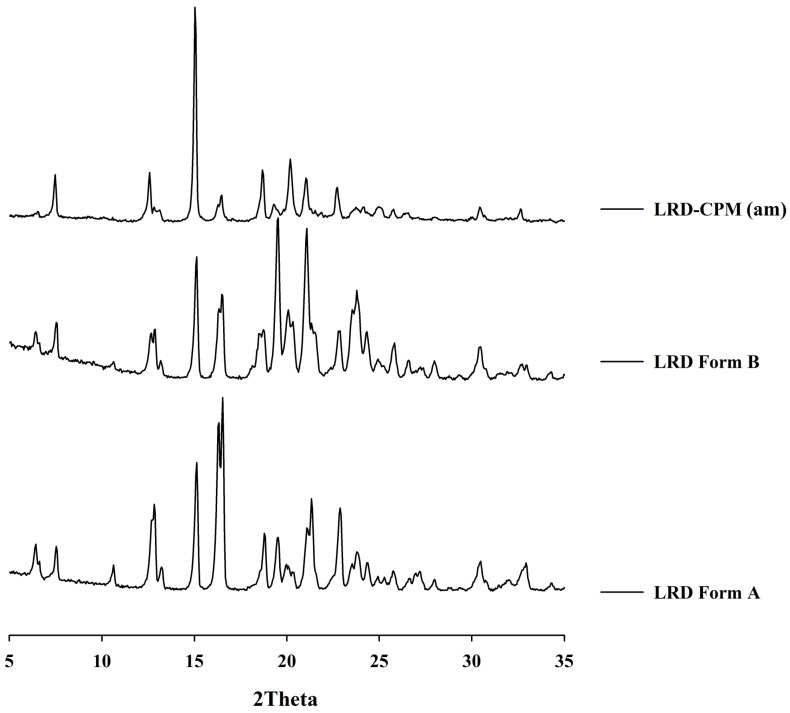
XRPD patterns of the co-amorphous mixture of LRD-CPM (am) after storage for 30 days in comparison with the intact polymorphic forms of LRD.

After all samples were kept in a desiccator cabinet at a temperature of 30 °C and 50% RH for 30 days, the XRPD patterns of some samples showed changes, as shown in Figure 3B. Crystalline peaks were observed in the XRPD pattern of co-amorphous LRD-CPM. The crystalline peaks detected at 2θ of 19.3, 20.2, and 24.1° were considered to belong to CPM. However, crystalline peaks at 2θ of 7.5, 12.6, 15.0, 16.2, 16.5, 18.7, 21.0, 22.7, 23.7, 30.4, and 32.6° were similar but not a complete match for the intact LRD. Two polymorphic forms (A and B) of LRD and their characteristics have been reported in US patent 20080194823A1 [29]. The intact LRD used in this study was in polymorphic form A. To clarify these crystalline peaks, LRD polymorphic form B was prepared according to a previous method [30]. The preparation was performed by slowly recrystallizing LRD from an amorphous LRD solution in a solvent mixture of methanol and water at a ratio of 20:80 for 10 h, after which it was filtered through a 0.22 µm filter membrane and dried in a desiccator at room temperature. The peak positions and intensities of the obtained recrystallized form B were compared with the crystalline peaks of the co-amorphous LRD-CPM stored for 30 days. The results showed that the peaks of the co-amorphous LRD-CPM matched those of LRD polymorphic form B, as shown in Figure 4.

**Figure 5:**
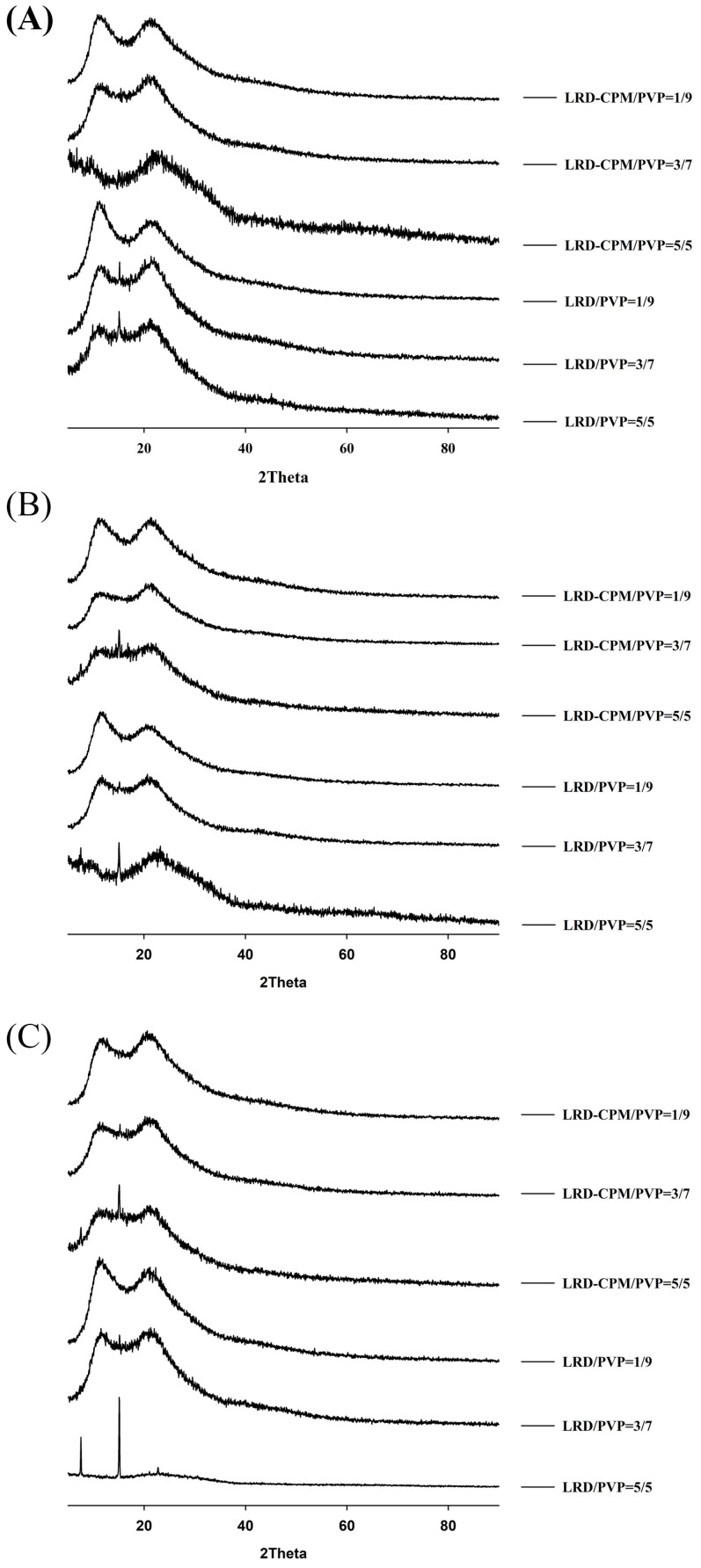
XRPD patterns of the amorphous LRD and co-amorphous LRD-CPM solid dispersions at 90 days (**A**), 180 days (**B**), and 360 days (**C**) of storage.

Keeping the samples for up to 360 days resulted in more changes, as shown in Figure 5. On the 90th day of storage, the crystalline peak of LRD at 2θ of 15.1° was observed for the LRD/PVP solid dispersions at ratios of 5/5 and 3/7, whereas the LRD-CPM/PVP solid dispersions at all ratios did not show any crystalline peaks, as shown in Figure 5A. On the 180th day of storage, the crystalline peak of LRD at 2θ of 15.1° started to emerge from the LRD-CPM/PVP solid dispersions at a ratio of 5/5, as shown in Figure 5B. After storage until day 360, the LRD-CPM/PVP solid dispersion at a ratio of 3/7 began to show the small crystalline peak of LRD at 15.1°. These results indicated that the solid dispersions of LRD-CPM/PVP possessed higher physical stability than the solid dispersion of LRD/PVP. In addition, the crystalline LRD formed in solid dispersions of LRD/PVP and LRD-CPM/PVP after storage was in the polymorphic form B.

### 3.5. FT-IR Analysis

**Figure 6:**
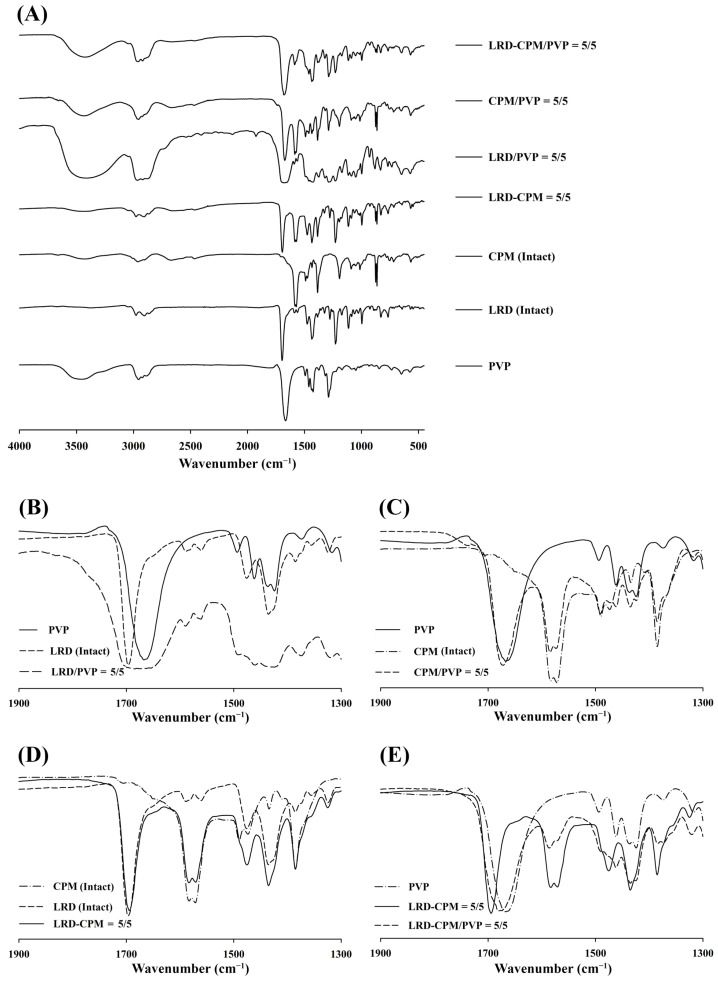
FT-IR spectra patterns of the co-amorphous LRD-CPM solid dispersions, CPM/PVP and LRD/PVP solid dispersions, and co-amorphous LRD-CPM mixture in comparison with the intact drugs and PVP (**A**) and wavenumber amplification in 1900–1300 cm^−1^ range of LRD/PVP solid dispersion (**B**), CPM/PVP solid dispersion (**C**), co-amorphous LRD-CPM mixture (**D**), and co-amorphous LRD-CPM/PVP solid dispersions (**E**).

FT-IR spectra were used to investigate the interactions between LRD, CPM, and PVP. The FT-IR spectra of all samples are shown in Figure 6. Due to the high hygroscopic nature of PVP, the broad -OH peak from moisture at 3500 cm^−1^ was also observed for PVP and all solid dispersions, as shown in Figure 6A. The spectra of LRD and PVP showed C=O peaks at 1696.5 and 1667.1 cm^−1^, respectively, as shown in Figure 6A. The spectra of CPM demonstrated peaks at 1583.1 and 1571.9 cm^−1^ assigned to C=C and C=N, respectively [31]. The prominent peaks observed in the region of 1600–1700 cm^−1^ can be used for assessing possible interactions between LRD, CPM, and PVP. The spectra of LRD/PVP and CPM/PVP solid dispersions are shown in Figure 6B,C, respectively, demonstrating C=O peaks at 1684.4 and 1672.3 cm^−1^, respectively. The carbonyl oxygen of PVP can act as a proton acceptor and participate in intermolecular interactions. However, there are no proton donors in the chemical structure of LRD and CPM; therefore, intermolecular interactions between PVP and LRD or PVP and CPM did not occur. Thus, the broad C=O peaks of the solid dispersions of LRD/PVP and CPM/PVP were the combination of both drugs and PVP. Furthermore, no peak shift was observed for the LRD-CPM co-amorphous mixture, as shown in Figure 6D, indicating that no intermolecular interactions occurred between LRD and CPM. As there was no intermolecular interaction between LRD, CPM, and PVP, there was no peak shift resulting from the intermolecular interaction for the co-amorphous LRD-CPM/PVP solid dispersion, as shown in Figure 6E.

### 3.6. Dissolution Studies

**Figure 7:**
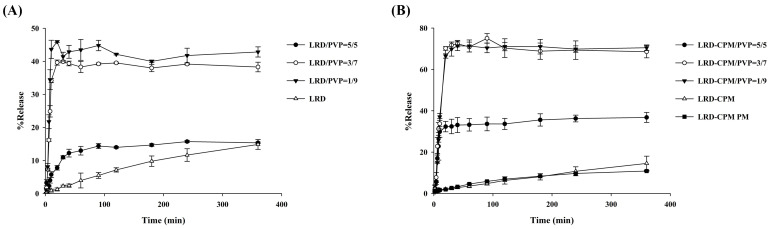
Dissolution profiles of LRD/PVP solid dispersions (**A**) and co-amorphous LRD-CPM/PVP solid dispersions (**B**) in comparison with intact LRD, co-amorphous LRD-CPM mixture, and LRD-CPM physical mixture in simulated intestinal fluid pH 6.8.

Dissolution profiles of intact LRD, co-amorphous LRD-CPM, solid dispersions of LRD/PVP and LRD-CPM/PVP, and the physical mixture of LRD-CPM in simulated intestinal fluid pH 6.8 are shown in Figure 7. Only 10–15% of LRD was released from the intact LRD and the physical mixture after 360 min. In contrast, the solid dispersions of both LRD/PVP and LRD-CPM/PVP significantly improved the dissolution of LRD. Surprisingly, co-amorphous LRD-CPM was unable to release large amounts of LRD despite LRD being in an amorphous state. This phenomenon could be explained by the rapid recrystallization process of LRD that occurred after exposure to the dissolution medium, which once again led LRD to have a low aqueous solubility. Solid dispersions of LRD/PVP and LRD-CPM/PVP could release LRD to a maximum amount of more than 40% and 70%, respectively, within 30 min. In addition, the initial dissolution rates of all solid dispersions were higher than those of intact LRD and the physical mixture. These results showed that improvement of LRD dissolution could be achieved using solid dispersions of drugs with PVP. They also suggested that solid dispersions could stabilize the amorphous state and inhibit the rapid recrystallization process of LRD. The amount of LRD released from the LRD-CPM/PVP solid dispersions was significantly higher than that from the LRD/PVP solid dispersions in the same drug-to-carrier ratio.

The release kinetic parameters and correlation coefficient (r^2^) of co-amorphous LRD-CPM, solid dispersions of LRD/PVP and LRD-CPM/PVP, and the physical mixture in the simulated intestinal fluid pH 6.8 for several release models were fitted to the approaching plateau curve at 90 min. For the Korsmeyer–Peppas equation, the first 60% of the release curve was used. All release kinetic parameters are shown in Table 3. It was found that most of the release kinetics of LRD from the co-amorphous LRD/PVP and solid dispersions of LRD/PVP and LRD-CPM/PVP best fit the Korsmeyer–Peppas equation. A release exponent (n) value of 0.5 indicates Fickian diffusion, 0.5 < n < 1.0 indicates anomalous diffusion, n = 1.0 indicates case II transport, and n > 1.0 indicates super case II transport [32]. The results indicated that LRD release from most systems is anomalous diffusion. The exponent characteristics (n) of the LRD-CPM/PVP solid dispersion increased as the amount of PVP increased, whereas the exponent characteristics of the LRD/PVP solid dispersion slightly decreased as the amount of PVP increased. It is known that the increase in exponent characteristics is attributed to the drug diffusion from the swelled matrix [33]. Thus, the increase in exponent characteristics of the LRD-CPM/PVP solid dispersion may be attributed to the high diffusion of LRD; when the matrix swelled, soluble additives of CPM and PVP were removed by dissolution, thus reducing the resistance of the gel layer to LRD diffusion.

## 4. Discussion

Solid dispersions are extensively used in drug development in response to dissolution problems. Several oral dosage form products using solid dispersion have been approved by the FDA in the last few decades [34,35]. Moreover, solid dispersions have been reported in several publications and global patents [36]. The solid dispersion of co-amorphous LRD-CPM with PVP exhibits improved dissolution and physical stability compared to intact LRD and the solid dispersion of LRD/PVP. It has been reported that the homogeneous combination of drugs with low-molecular-weight molecules in the amorphous state results in high drug solubility [37]. This study improved the solubility of LRD in the co-amorphous state by incorporating co-amorphous LRD-CPM with PVP as a hydrophilic polymer. Due to the high energy of the amorphous state and the lack of energy required for the crystal lattice rearrangement during dissolution, co-amorphous substances, therefore, have great drug solubility. In general, the solubility of poorly soluble drugs tends to increase with the number of water-soluble polymers in a solid dispersion [38]. In this study, as the amount of PVP was increased, the solubility of LRD was significantly increased due to increased wettability and dispersibility of the drug by hydrophilic carriers. In DSC thermograms, Tg can be used to describe drug–drug interactions or polymer chain mobility due to drug–polymer interaction. The single-phase co-amorphous system reflected in the DSC thermograms with a single Tg indicates the miscibility of the co-amorphous system, which corresponds to the temperature of the transformation of the amorphous material from the glass state to a viscous fluid. The deviation in Tg in a binary mixture could be due to the weight fraction of each component of a drug–polymer system. The decrease in Tg in a binary mixture of drug and PVP as a function of drug loading was also reported [39,40]. The increase in Tg of the co-amorphous system is likely to be the major component in the system. Although the Tg of the co-amorphous components is lower than that of the individual amorphous components, an amorphous system is not the most stable system [41]. The results in the current study show that Tg is not the only factor affecting the stability of the co-amorphous systems. In addition, we have compared our results with similar work previously reported and found that the results are in good agreement [42]. A high content of PVP can give a high degree of an amorphous drug.

The amorphous state of the LRD-CPM solid dispersion shows a broad halo pattern without sharply intense peaks for at least 360 days of storage. Amorphous stability, which depends on the polymer in a solid dispersion, is an important factor in improving the dissolution rate and solubility. The crystallization of an amorphous drug in a solid dispersion with polymorph transformation has been reported [43,44]. Although the slow crystallization of LRD from a co-amorphous mixture occurs rapidly, adding PVP can keep amorphousness stable for up to 360 days. FT-IR was extensively used to identify the drug–polymer interaction with the obviously observed peak shift if the interactions between the drug and polymer occurred. Without drug and polymer interactions, only minor or no changes in the FT-IR spectra were observed, as previously reported [45]. Our results show that the co-amorphous solid dispersion of LRD-CPM with PVP leads to enhanced stability even in the absence of interaction due to molecular mixing. Stable solid dispersion systems without drug–carrier interactions have also been reported [46,47]. In addition, our results also show that dissolution is enhanced by adding PVP to co-amorphous LRD-CPM.

## 5. Conclusions

In conclusion, this study demonstrates the development and evaluation of co-amorphous LRD-CPM/PVP solid dispersions prepared by the film casting method. The solid-state characterization results of DSC, PXRD, and FT-IR showed an amorphous form of LRD without intermolecular interactions between LRD, CPM, and PVP. Increasing the PVP ratio extended the amorphous state of LRD in both LRD/PVP and LRD-CPM/PVP during storage. The co-amorphous LRD-CPM/PVP solid dispersion exhibited a synergistic interaction that stabilized the amorphous state of LRD compared to LRD/PVP. In addition, polymorphic transformation of LRD was observed due to the slow crystallization of LRD. The solubility and dissolution of LRD-CPM/PVP were higher than those of LRD/PVP and intact LRD. The release mechanisms of both the solid dispersions of LRD/PVP and LRD-CPM/PVP can be described by the Korsmeyer–Peppas model, showing that the drug release occurs from a swollen matrix. In this respect, the co-amorphous solid dispersion of LRD-CPM using PVP as a carrier presents the advantages of high solubility and high dissolution of LRD and amplification of the amorphous state of LRD. Co-amorphous solid distributions may be a future challenge for drug combination development to improve the properties of drugs and to increase their effectiveness.

## Figures and Tables

**Table 1:** Solubility of amorphous LRD and co-amorphous LRD-CPM solid dispersions at 48 h in simulated intestinal fluid pH 6.8.

Samples	Solubility * (µg/mL)	
	Physical Mixture	Solid Dispersion
LRD/PVP = 5/5	4.30 ± 0.86	7.97 ± 0.33
LRD/PVP = 3/7	4.95 ± 1.10	17.60 ± 5.24
LRD/PVP = 1/9	13.84 ± 1.76	37.63 ± 7.41
LRD-CPM/PVP = 5/5	5.71 ± 0.42	62.37 ± 16.96
LRD-CPM/PVP = 3/7	13.87 ± 7.85	84.47 ± 12.21
LRD-CPM/PVP = 1/9	12.34 ± 7.78	195.03 ± 13.44

* Solubility of LRD (intact) is 4.71 ± 1.19 mg/mL.

**Table 2:** Glass transition temperature (Tg) of amorphous LRD and co-amorphous LRD-CPM solid dispersions.

Samples	Tg * (°C)
LRD/PVP = 5/5	158.17
LRD/PVP = 3/7	179.50
LRD/PVP = 1/9	184.72
LRD-CPM/PVP = 5/5	150.70
LRD-CPM/PVP = 3/7	154.98
LRD-CPM/PVP = 1/9	172.33

* Tg of PVP (intact) is 182.58 °C.

**Table 3:** Release kinetic parameters (k) and correlation coefficients (r^2^) of amorphous LRD and co-amorphous LRD-CPM solid dispersions in simulated intestinal fluid pH 6.8, in comparison with the intact drugs and their physical mixture.

Sample	First-Order		Higuchi		Korsmeyer–Peppas			Hixson–Crowell	
	k	r^2^	k	r^2^	n	k	r^2^	k	r^2^
LRD (intact)	0.0007	0.808	0.464	0.734	0.62	0.241	0.763	0.0002	0.806
CM (intact)	0.0006	0.249	0.487	0.827	0.28	0.937	0.823	0.0002	0.244
LRD/PVP = 5/5	0.0024	0.575	1.675	0.908	0.68	1.025	0.925	0.0008	0.555
LRD/PVP = 3/7	0.0109	0.102	5.737	0.516	0.49	7.667	0.778	0.0031	0.242
LRD/PVP = 1/9	0.0137	0.250	6.561	0.363	0.42	1.082	0.642	0.0038	0.420
LRD-CPM/PVP = 5/5	0.0084	0.322	4.902	0.445	0.44	7.451	0.708	0.0025	0.439
LRD-CPM/PVP = 3/7	0.0377	0.832	9.984	0.766	0.66	7.365	0.889	0.0111	0.763
LRD-CPM/PVP = 1/9	0.0339	0.824	9.593	0.760	0.71	5.904	0.886	0.0100	0.756
Physical mixture = 5/5	0.0008	0.775	0.558	0.924	0.44	0.606	0.805	0.0003	0.771

## Data Availability

Not applicable.
